# Association Between the Dietary Index for Gut Microbiota (DI-GM) and Colorectal Cancer in the PLCO Cohort

**DOI:** 10.3390/nu18071088

**Published:** 2026-03-28

**Authors:** Bezawit E. Kase, Angela D. Liese, Jiajia Zhang, Elizabeth Angela Murphy, Susan E. Steck

**Affiliations:** 1 Department of Epidemiology & Biostatistics, Arnold School of Public Health, University of South Carolina, Columbia, SC 29208, USA; kasebe@dph.sc.gov (B.E.K.); liese@mailbox.sc.edu (A.D.L.); jzhang@mailbox.sc.edu (J.Z.); 2 Department of Pathology, Microbiology & Immunology, School of Medicine Columbia, University of South Carolina, Columbia, SC 29209, USA; angela.murphy@uscmed.sc.edu

**Keywords:** dietary index for gut microbiota, colorectal cancer, cancer, diet, gut microbiome

## Abstract

Objectives: The study aimed to examine the association between a dietary index for gut microbiota (DI-GM) and the risk of incident colorectal cancer (CRC). Clarifying the role of diet-induced alterations in the composition and function of gut microbiota on the development of CRC can contribute to prevention efforts. Methods: Participants from the Prostate, Lung, Colorectal, and Ovarian Cancer Screening trial enrolled in the intervention arm and who completed baseline assessments were included in the analysis (*n* = 55,685). The DI-GM is a literature-derived index used to score diet quality in terms of maintaining healthy gut microbiota. A time-dependent Cox model stratified by follow-up years (<5 and ≥5 person-years) was used to evaluate the relationships between the dietary patterns and risk of incident CRC. Results: A total of 735 incident CRC were identified over 650,470 person-years of follow-up. During < 5 years of follow-up, those with higher diet quality (DI-GM scores above 67th percentile) had an 18% lower risk of incident CRC (HR_adjusted_ = 0.82, 95% CI: 0.63, 1.07) compared with those with lower diet quality (DI-GM scores below the 67th percentile), though effect estimates were imprecise. During ≥ 5 years of follow-up, there was no association between incident CRC and DI-GM (HR_adjusted_ = 1.01, 95% CI: 0.80, 1.26). Conclusions: Diet quality measured using the DI-GM was associated with the risk of CRC in the first five years of follow-up in a large prospective cohort study. A diet that enhances the composition and function of gut microbiota may contribute to reduction in CRC risk.

## 1. Introduction

Colorectal cancer (CRC) is ranked as the third most diagnosed cancer and the second leading cause of cancer-related deaths worldwide [1]. The gut microbiota has a role in the development of CRC by mechanisms that lead to aberrant activation of the immune system resulting in chronic inflammation and increased cellular proliferation [2]. The contribution of the gut microbiota to the initiation and progression of CRC presents a potential avenue for preventive strategies to curb the burden of CRC [3]. To this end, there is a growing interest in identifying factors that can modify the gut microbiota and thereby reduce the risk of CRC.

Around 90% of CRC occurs without a family history or genetic predisposition [3], indicating a considerable potential to reduce the risk of CRC through modifiable factors such as diet [4]. The effect of food groups and dietary patterns on the risk of CRC has been investigated [5]. However, the evidence regarding the effect of diet on the risk of CRC within the context of gut microbiota is scarce. A few studies examined diet’s role in gut bacteria-induced CRC mechanisms [6,7,8,9]. Nguyen et al. demonstrated that diet-induced alterations in sulfur-metabolizing microbes could modify CRC risk using a sulfur microbial diet score which was derived using reduced rank regression [8,9]. Although this diet score is informative, it is limited to sulfur-metabolizing bacterial species and consequently examines one mechanism: an increase in the microbial production of hydrogen sulfide, while other cancer-associated taxa such as Fusobacterium are not addressed. Another study by Okeefe et al. focused on the effect of specific nutrients rather than overall diet on gut microbiota-related risk of CRC [10]. Thus, additional tools are needed to investigate whether diet-induced alteration of composition and function of gut microbiota can impact the development of CRC.

In a prior investigation, we derived a dietary index for gut microbiota (DI-GM) using a systematic review of literature [11]. Foods and nutrients linked with the composition and function of the gut microbiota were used to build the DI-GM. Using data from the National Health and Nutrition Examination Survey (NHANES) in the United States, we found that a higher score on DI-GM, which indicates higher diet quality, was associated with greater gut microbiota diversity. To date, only one previous study has examined associations between the DI-GM and CRC, reporting higher DI-GM scores were associated with lower risk of CRC within the United Kingdom Biobank [6].

To further investigate whether diet-induced alterations in the composition and function of gut microbiota can affect the development of CRC, we examined the association between the DI-GM and the risk of CRC. This is an observational study using secondary data from a large prospective study embedded within a clinical trial in the United States. In addition, we examined the association between other dietary indices (Healthy Eating Index (HEI-2020) and Mediterranean Diet Score (MDS)) and the risk of CRC and compared these associations with the DI-GM association.

## 2. Materials and Methods

### 2.1. Study Population

The Prostate, Lung, Colorectal and Ovarian Cancer Screening Trial (PLCO) is a multicenter, two-armed, randomized trial designed by the National Cancer Institute (NCI, United States) to determine the effects of screening on cancer prognosis and cancer-related mortality [12,13,14]. Eligible individuals were aged 55–74 years, had no history of prostate, lung, colorectal or ovarian cancer, were not undergoing cancer treatment, and not currently participating in another cancer related trial [13]. The intervention arm received screening tests at entry, then annually for five years, and the control arm followed usual medical care practices. Both arms were followed for at least 13 years from randomization to ascertain all the prostate, lung, colorectum, and ovarian cancers and deaths from all causes. The PLCO trial was approved by the Institutional Review Boards (IRB)at the NCI, approved as exempt by the University of South Carolina IRB (STUDY ID #00000668), and was registered with the United States National Institutes of Health as a clinical trial (www.ClinicalTrials.gov URL accessed on 17 March 2021; [NCT01696981—for colorectal cancer]).

For the present study, participants enrolled in the intervention arm who had no personal history of cancer and who completed baseline assessments were included in the analysis. As recommended by the PLCO study team, we did not include participants in the control arm due to a difference in the food frequency questionnaire (FFQ) and timing of administration of the FFQ in that group. Participants were excluded if they had missing data on sociodemographic characteristics (n = 235), family history of CRC (n = 407), body mass index (BMI) (n = 554), regular use of aspirin and nonsteroidal anti-inflammatory drugs (NSAIDs) (n = 184), or if they had implausible energy intake (<800 or >4200 kcal/day for males and <600 or >3500 kcal/day for females). In total, 55,685 participants comprised our final study population.

### 2.2. Dietary Assessment

Dietary intake among participants in the intervention arm was measured using the Diet Questionnaire (DQX), a self-administered 137-item FFQ that inquired about usual dietary intake over the preceding year at baseline of the PLCO trial. Reported mixed foods were disaggregated into cup equivalents foods and food groups using the Food Patterns Equivalents Database from the U.S. Department of Agriculture. The disaggregated dietary data were used to compute the DI-GM, Healthy Eating Index-2020 (HEI-2020) and Mediterranean Diet Score (MDS).

The DI-GM is a dietary index that scores diet based on effects on the composition and function of gut microbiota [11]. The DI-GM has 14 components: fermented dairy, chickpeas, soybean, whole grains, refined grains, fiber, cranberries, avocados, broccoli, red meat, processed meat, high-fat diet (≥40% energy from fat), coffee, and green tea. Each component in the DI-GM is scored a 0 or 1 based on the sex-specific median intake values. A score of 1 was assigned for each component if a participant consumed above the sex-specific median for fermented dairy, chickpeas, soybean, whole grains, fiber, cranberries, avocados, broccoli, coffee, and green tea; otherwise, a score of 0 was assigned. A score of 1 was assigned for each component if a participant consumed below the sex-specific median for refined grains, red meat, processed meat, and a high-fat diet (≥40% energy from fat); otherwise, a score of 0 was assigned. The DI-GM is the sum of the scores for each component and can range from 0–14. In the PLCO, soybean, whole grains, refined grains, fiber, red meat, processed meat, broccoli, high fat diet (≥40% energy from fat) and coffee were included in the disaggregated dietary data. The fermented dairy, chickpeas, cranberries, avocados, and green tea components were not included in the disaggregated dietary data. We computed fermented dairy component by taking the sum of intake of yogurt, cheese and sour cream. We used legumes in place of chickpeas; citrus, melons, and berries fruit group in place of cranberries; and other fruit group in place of avocados. Green tea was not included in the DQX, thus the score for the DI-GM ranged from 0–13. The DI-GM was categorized into tertiles in the main analysis, with the highest third tertile indicating higher diet quality.

We computed the HEI-2020 and the MDS. The HEI-2020 has 13 components: total fruit, whole fruit, total vegetables, greens and beans, whole grains, dairy, total protein, seafood and plant proteins, polyunsaturated (PUFA) + monounsaturated (MUFA)/saturated (SFA), refined grains, sodium, added sugars, and saturated fats [15,16]. The HEI-2020 total score ranges from 0 to 100, with higher scores indicating better conformity with the Dietary Guidelines for Americans, 2020. The MDS has nine components: vegetables, legumes, fruits and nuts, cereals, fish, dairy products, meat and poultry, the ratio of monounsaturated to saturated fatty acids and alcohol [17]. The total score for the MDS ranges from 0 to 9 with higher scores indicating better conformity to the Mediterranean diet. Food components and scoring algorithms for the DI-GM, HEI-2020, and MDS can be found in Supplementary Materials Tables S1–S3.

### 2.3. Colorectal Cancer Ascertainment

Incident CRC was identified primarily through annual health update questionnaires and screening conducted at either 3 or 5 years after baseline. Diagnoses of CRC and tumor characteristics were confirmed through medical record review. The person-years were measured from completion of the baseline dietary assessment (trial entry) to the earliest date of CRC diagnosis, death, or censoring.

### 2.4. Covariates Assessment

At baseline, sociodemographic characteristics, health-related behaviors, medical history, and cancer risk factors were assessed. This included age at randomization (years), sex (male, female), race (non-Hispanic White, non-Hispanic Black, Hispanic, Asian, others), education (<high school, high school graduate and some college, college graduate, or postgraduate), marital status (married, widowed/divorced/separated, never married), occupation (homemaker, working, unemployed/retired), body mass index (in kg/m^2^), alcohol use (abstainer, 0–7 drinks/week, >7 drinks/week), smoking (never, former smoker with <18 pack-years, former smoker with 18–41 pack-years, former smoker with ≥41 pack-years, current smoker with <18 pack-years, current smoker with 18–41 pack-years, current smoker with ≥41 pack-years), regular aspirin or NSAID use (yes, no), and family history of CRC (no, probably or yes). Physical activity (number of days of vigorous physical activity per week) was assessed using a supplemental questionnaire that was added between 2006 and 2008.

### 2.5. Statistical Analysis

Cox proportional hazard models were used to estimate the age and total energy intake-adjusted and multivariable-adjusted hazard ratios (HR) and 95% confidence intervals (CI) of incident CRC associated with DI-GM categories. Analogous analyses were run to examine association between HEI-2020 and MDS and risk of CRC.

Schoenfeld residual test was used to check violations of the proportional hazard assumption for all covariates in the models [18]. The proportional hazards assumption was not met for DI-GM (tertiles), sex, and regular aspirin or NSAIDs use. A time-dependent Cox model stratified by follow-up years (<5 and ≥5 person-years), sex, and regular aspirin or NSAIDs use was ran, and interaction assumption was assessed using likelihood ratio test. The 5 years of follow-up cutoff was selected based on the survival probability curve which showed the curves for the DI-GM categories were not parallel at 5 years of follow-up. The assumption of no interaction was met for sex and regular aspirin or NSAIDs use. Separate HRs with 95% confidence intervals were reported for <5 and ≥5 person-years of follow-up. The HRs and 95%CIs were calculated for both DI-GM categories and the continuous DI-GM score.

In sensitivity analysis, we additionally adjusted for physical activity in the multivariable model using a sub-sample that had completed the supplemental questionnaire containing physical activity information (n = 40,165). We also analyzed the association between the DI-GM and the risk of incident CRC by anatomical subsites (proximal and distal) and by sex. All statistical tests were two-sided at α = 0.05 and analyses were performed using SAS^®^ 9.4 software.

## 3. Results

There were 735 incident cases of CRC over 650,470 person-years of follow-up. The median years of follow-up was 12.2 years. The mean ± SD age of participants was 62.6 ± 5.32 years and around half (50.7%) of the participants were males. The majority of the participants were non-Hispanic White (90.9%), married (78.4%), and had no family history of CRC (86.8%). The mean ± standard deviation (SD) for the DI-GM score was 6.99 ± 2.34, with a range of 0 to 13. The mean ± SD for the HEI-2020 score was 66.8 ± 8.86 and for the MDS score was 4.23 ± 1.54. Baseline characteristics across strata of DI-GM tertiles are shown in Table 1.

The Kaplan-Meier survival curves for incident CRC by DI-GM tertiles are presented in Supplementary Materials Figure S1. The survival curves for participants in the first and second DI-GM tertiles were similar. Thus, these two DI-GM tertiles were collapsed into one group and the HRs were estimated for the third DI-GM tertile (above 67th percentile) compared to the first and second DI-GM tertiles (below 67th percentile).

HRs for incident CRC risk stratified by follow-up time (<5 and ≥5 person-years in the time-dependent Cox models) are presented in Table 2. During <5 years of follow-up, those who scored above the 67th percentile of the DI-GM (higher diet quality) had a 26% lower risk of incident CRC compared with those who scored below the 67th percentile of the DI-GM (lower diet quality) after adjusting for age and total energy intake (HR = 0.74, 95% CI: 0.57, 0.95). In the multivariable analyses, the effect estimate was slightly attenuated and no longer statistically significant (HR = 0.82, 95% CI: 0.63, 1.07 comparing above to below the 67th percentile of the DI-GM). After excluding BMI from the multivariable analyses, results were similar to the model with BMI; those who scored above the 67th percentile of the DI-GM had a 20% lower risk of incident CRC compared with those who scored below the 67th percentile of the DI-GM in the first five years of follow-up (HR = 0.80, 95% CI: 0.62, 1.04).

During ≥5 years of follow-up, there were no differences in risk of incident CRC between those who scored above the 67th percentile of the DI-GM and those who scored below the 67th percentile of the DI-GM after adjusting for age and total energy intake (HR = 0.90, 95% CI: 0.72, 1.12) or in the multivariable analyses (HR = 1.01, 95% CI: 0.80, 1.26).

During <5 years of follow-up, those who scored above the 67th percentile of HEI-2020 had a 13% lower risk of incident CRC compared to those who scored below the 67th percentile of HEI-2020 (HR = 0.87, 95% CI: 0.68, 1.11) in the multivariable analyses. During ≥5 years of follow-up, the association for the HEI-2020 was null (HR = 1.00, 95% CI: 0.81, 1.24).

In the multivariable analyses, those who scored above the 67th percentile of the MDS had a 20% lower risk of incident CRC compared to those who scored below the 67th percentile of the MDS (HR = 0.80, 95% CI: 0.64, 0.99) in the first five years. There were no differences in risk of incident CRC between the two groups after five years of follow-up (HR = 1.12, 95% CI: 0.92, 1.37).

All results from the sub-group and sensitivity analyses are shown in Supplementary Materials Tables S4–S6. In the first five years of follow-up, the association between the DI-GM and risk of CRC was slightly stronger in females (HR = 0.72, 95% CI: 0.46, 1.13) than males (HR = 0.88, 95% CI: 0.64, 1.22). However, we found no evidence of interaction by sex (p_interaction_ = 0.27). The association was also slightly stronger in distal (HR = 0.79, 95% CI: 0.55, 1.12) than proximal (HR = 0.89, 95% CI: 0.63, 1.07) CRC in the first five years. In the sensitivity analysis with physical activity included in the multivariable model, results were similar to the original multivariable models.

## 4. Discussion

A literature-derived dietary index that captures the relationship between diet and gut microbiota composition and function was used to examine the association between diet quality and the risk of CRC. In the first five years of follow-up, higher diet quality indicated by a higher DI-GM score was associated with a lower risk of incident CRC though effect estimates were imprecise. After five years of follow-up, we found no association between the DI-GM and the risk of CRC. We found similar associations between other dietary indices (HEI-2020 and MDS) and the risk of incident CRC.

We found that adherence to a diet that enhances gut microbiota composition and function is associated with a modest lower risk of CRC. In line with the current findings, a study based on the UK Biobank found a significant association between adherence to high DI-GM diet and reduced risk of CRC in which participants completed multiple dietary assessments over 13-years of follow-up [6]. A prior study demonstrated lower adherence to sulfur-microbial diet (high diet quality) was associated with a 12% lower risk of CRC [19]. Other investigations on specific nutrients, i.e., fat and fiber, have suggested that the effect of diet on the risk of CRC may be mediated by microbiota and their metabolites [20,21]. The current study contributes to the growing evidence on the use of diet to modulate gut microbiota, thereby lowering the risk of CRC.

The use of a single dietary assessment in the current study may have contributed to the absence of association between the DI-GM and the risk of CRC after the first five years of follow-up. Given the study has an extended follow-up time, there is a potential for intraindividual variability in diet over time. Participants in PLCO trial were aged 63 years on average at the start of the study, and as participants age, they may consume a more beneficial diet that may reverse dysbiosis. For instance, a previous study has shown older adults tend to consume less energy-dense sweets and fast foods, and consume more nutrient-dense grains, vegetables, and fruits [22]. In addition, substantial dietary changes in adults aged 50–65 years can result in gut microbiota changes that enhance butyrogenesis, lower mucosal proliferation and inflammation, all of which may reduce the risk of the developing CRC [7]. To evaluate the effect of long-term usual diet, multiple measures of diet over the follow-up years that captures intraindividual variation in diet would have been more informative. However, in the PLCO two dietary assessments were conducted 5 years apart using different tools which did not allow for construction of aggregate dietary scores over time.

The foods and nutrients included in the DI-GM fit in the context of prior work that has demonstrated the relationship between diet and risk of CRC. The WCRF/AICR report showed that red meat and processed meat are probable and convincing causes of CRC, respectively [23]. In addition, there is strong evidence that consuming whole grains and fiber reduces the risk of CRC [23]. There is some evidence that consuming broccoli, soy, and coffee are inversely associated with CRC risk [21,24,25]. Although evidence exists for associations involving individual foods and nutrients, the use of a dietary index is advantageous because it accounts for combinations of biological effects of different dietary factors and reduces multiple testing.

The association between diet and risk of CRC was similar across the different dietary indices, i.e., DI-GM, HEI-2020, and MDS, used in this study. Previous studies that used HEI and MDS have shown an inverse association with the risk of CRC [5]. We anticipated a stronger association between DI-GM and risk of CRC as compared to using HEI-2020 or MDS and risk of CRC. The lack of stronger association may be due to not having some of the specific food components of the DI-GM in the PLCO dataset (e.g., green tea) and having to substitute for more general food groups (e.g., legumes instead of chickpeas). Other studies that use open-ended dietary assessment are needed to examine the full utility of the DI-GM.

This study expands on prior research on the relationship between diet and CRC by using a novel *a priori* dietary index that incorporates the role of gut microbiota in the diet-CRC relationship. The use of a large cohort with an extended follow-up time is a strength and the PLCO captured data on multiple potential confounders which were included in the analyses. However, this study has some limitations that ought to be considered while interpreting the findings. In addition to the misclassification of dietary intakes from self-reported data [26], the use of close-ended questions in FFQ was not sufficient to score all components of the DI-GM. The lack of multiple dietary assessments did not allow the capture of variability of diet over time. The generalizability of the findings may be limited to the older non-Hispanic White population. Loss to follow up may present a potential for selection bias, however the response rate in the PLCO was relatively high at 84%, suggesting this may not have impacted results substantially. Finally, it is possible that the stronger association observed in the first five years of follow-up may be related to reverse causation whereby individuals may have changed their diet due to early symptoms of disease. However, the impact of this is likely to be minimal given that all participants were screened at baseline for CRC and were asymptomatic for the PLCO target cancers at baseline.

## 5. Conclusions

The DI-GM, which was developed based on the effects of diet on gut microbiota, was associated with the risk of CRC during the first five years of follow-up but not thereafter. These findings add to the growing evidence that diet-induced alterations in the composition and function of the gut microbiota may be linked to CRC development. Future studies with larger and diverse cohorts, using open-ended dietary assessments (e.g., 24-h dietary recalls) and repeated measurements over time, are needed to further substantiate this association.

## Figures and Tables

**Table 1:** Characteristics of PLCO study participants across the DI-GM tertiles.

	Total Sample	DI-GM Tertiles (Score Ranges)		
		1st (0–5)	2nd (6–8)	3rd (9–13)
n	55,685	15,551	25,066	15,068
Person-years	650,469.6	175,597.5	292,582.0	182,290.1
Colorectal cancer cases, n (%)				
Total	735 (1.3)	197 (1.3)	348 (1.4)	190 (1.3)
Proximal colon	307 (41.8)	111 (56.4)	194 (55.7)	120 (63.2)
Distal colon	425 (57.8)	86 (43.7)	151 (43.4)	70 (36.8)
Unclear	3 (0.4)	0	3 (0.90)	0
DI-GM score, (mean ± SD)	6.99 ± 2.34	4.12 ± 1.00	6.99 ± 0.81	9.93 ± 1.00
HEI-2020 (mean ± SD)	66.8 ± 8.86	59.9 ± 8.23	67.1 ± 7.29	73.3 ± 6.37
MDS (mean ± SD)	4.23 ± 1.54	3.23 ± 1.31	4.20 ± 1.34	5.32 ± 1.32
Age at randomization, (mean ± SD)	62.6 ± 5.32	61.7 ± 5.15	62.7 ± 5.31	63.4 ± 5.38
Sex, n (%)				
Male	28,242 (50.7)	7464 (48.0)	12,764 (50.9)	8014 (53.2)
Female	27,443 (49.3)	8087 (52.0)	12,302 (49.1)	7054 (46.8)
Race and ethnicity, n (%)				
White, Non-Hispanic	50,623 (90.9)	14,104 (90.7)	22,801 (91.0)	13,709 (91.0)
Black, Non-Hispanic	2083 (3.7)	737 (4.7)	876 (3.5)	470 (3.1)
Hispanic	823 (1.5)	266 (1.7)	355 (1.4)	202 (1.3)
Asian	1804 (3.2)	327 (2.1)	864 (3.5)	613 (4.1)
Other	352 (0.6)	117 (0.8)	161 (0.6)	74 (0.5)
BMI (kg/m^2^, mean ± SD)	27.2 ± 4.78	28.1 ± 5.13	27.3 ± 4.67	26.3 ± 4.40
Total energy intake (kcal/day; mean ± SD)	1990.0 ± 706.1	1804.9 ± 671.4	1992.6 ± 726.7	2176.5 ± 654.6
Number of days of vigorous PA per week, n (%)				
None or <1	20,842 (51.9)	6500 (58.9)	9432 (52.1)	4910 (44.6)
2–3	13,519 (33.7)	3349 (30.3)	6112 (33.8)	4058 (36.8)
4–5	4250 (10.6)	891 (8.07)	1883 (10.4)	1476 (13.4)
6–7	1554 (3.87)	303 (2.74)	679 (3.8)	572 (5.2)
Alcohol use, n (%)				
Abstainer	9953 (17.9)	3254 (20.9)	4389 (17.5)	2310 (15.3)
0–7 drinks/week	33,019 (59.3)	8926 (57.4)	14,848 (59.2)	9245 (61.4)
>7 drinks/week	12,713 (22.8)	3371 (21.7)	5829 (23.3)	3513 (23.3)

n—sample size, %—percentage, ±SD—standard deviation, DI-GM—dietary index for gut microbiota, HEI-2020—healthy eating index, MDS—Mediterranean diet score, BMI—body mass index, PA—physical activity.

**Table 2:** Association between baseline dietary quality and incidence of colorectal cancer, PLCO.

Dietary Quality	Number of Cases	Person-Years	Model 1 ^a^	Model 2 ^b^	Model 3 ^c^
**DI-GM** (High versus low quality) ^d^					
First 5 years of follow-up	330	6631	0.74 (0.57, 0.95) *	0.82 (0.63, 1.07)	0.80 (0.62, 1.04)
After 5 years of follow-up	405	643,838	0.90 (0.72, 1.12)	1.01 (0.80, 1.26)	0.97 (0.78, 1.22)
Continuous **DI-GM** score					
First 5 years of follow-up			0.94 (0.90, 0.99) *	0.97 (0.92, 1.01)	0.96 (0.91, 1.01)
After 5 years of follow-up			0.97 (0.93, 1.02)	1.00 (0.96, 1.05)	0.99 (0.95, 1.04)
**HEI-2020** (High versus low quality) ^d^					
First 5 years of follow-up	330	6631	0.75 (0.59, 0.95) *	0.87 (0.68, 1.11)	0.84 (0.66, 1.08)
After 5 years of follow-up	405	643,838	0.92 (0.75, 1.13)	1.00 (0.81, 1.24)	0.97 (0.79, 1.20)
**MDS** (High versus low quality) ^d^					
First 5 years of follow-up	330	6631	0.76 (0.61, 0.95) *	0.80 (0.64, 0.99) *	0.78 (0.62, 0.98) *
After 5 years of follow-up	405	643,838	1.06 (0.87, 1.29)	1.12 (0.92, 1.37)	1.10 (0.90, 1.34)

DI-GM—dietary index for gut microbiota, HEI-2020—healthy eating index, MDS—Mediterranean diet score; ^a^ Adjusted for age and total energy intake; ^b^ Adjusted for variables in a and sex, race, education, marital status, smoking, alcohol use, BMI, regular aspirin or NSAID use, family history of CRC; ^c^ Adjusted for variables in b but excluding BMI; ^d^ High quality (above 67th percentile) versus low quality (below 67th percentile), * *p* < 0.05.

## Data Availability

This study was conducted using secondary data analyses of the PLCO Cancer Screening Trial. No new data were created or analyzed in this study. Data sharing is not applicable to this article.

## Supplementary Materials

The following supporting information can be downloaded at: https://www.mdpi.com/article/10.3390/nu18071088/s1, Table S1: Components and scoring of the Dietary Index for Gut Microbiota (DI-GM) in the PLCO dataset; Table S2: Component and scoring of the Healthy Eating Index-2020 (HEI-2020) for ages 2 and over; Table S3: Component and scoring of the Mediterranean Diet Score (MDS); Table S4: Subgroup analysis of the relationship between dietary indices and colorectal cancer by sex in PLCO; Table S5: Subgroup analysis of the relationship between dietary indices and colorectal cancer by location of cancer in PLCO; Table S6: Sensitivity analysis of the relationship between dietary indices and colorectal cancer in a sub-sample of PLCO participants with physical activity data; Figure S1: Kaplan-Meier plots of colorectal cancer incidence by DI-GM tertiles in PLCO Study.
