# Supplementary material for: Association Between the Dietary Index for Gut Microbiota (DI-GM) and Colorectal Cancer in the PLCO Cohort
Source: Nutrients. 2026 Mar 28;18(7):1088. doi: 10.3390/nu18071088 (PMC13074818; doi:10.3390/nu18071088)
Supplement: Supplementary file 1 [file nutrients-18-01088-s001.zip › nutrients-4163606-supplementary.pdf]

Supplementary Materials

Table S1. Components and scoring of the Dietary Index for Gut Microbiota (DI-GM) in the PLCO dataset

No.	Component	Application in the PLCO dataset	Beneficial/harmful to gut microbiota	Scoring
1	Fermented dairy	Intake of yogurt, cheese and sour cream	Beneficial	1 if consumption at or above the sex-specific median, else 0
2	Red meat	Beef, veal, pork, lamb, and game meat; excludes organ meat and cured meat	Harmful	0 if consumption at or above the sex-specific median, else 1
3	Processed meat	Frankfurters, sausages, corned beef, and luncheon meat that are made from beef, pork, or poultry	Harmful	0 if consumption at or above the sex-specific median, else 1
4	Chickpea	Intake of Legumes	Beneficial	1 if consumption at or above the sex-specific median, else 0
5	Soybean	Soy products - Soy milk, Tofu	Beneficial	1 if consumption at or above the sex-specific median, else 0
6	Whole grains	Grains defined as whole grains and contain the entire grain kernel —the bran, germ, and endosperm	Beneficial	1 if consumption at or above the sex-specific median, else 0
7	Refined grains	Refined grains that do not contain all of the components of the entire grain kernel	Harmful	0 if consumption at or above the sex-specific median, else 1
8	Fiber		Beneficial	1 if consumption at or above the sex-specific median, else 0
9	Cranberries	Citrus, melons, and berries	Beneficial	1 if consumption at or above the sex-specific median, else 0
10	Avocados	Other fruit	Beneficial	1 if consumption at or above the sex-specific median, else 0
11	Broccoli	Broccoli	Beneficial	1 if consumption at or above the sex-specific median, else 0
12	High fat diet (% energy)		Harmful	0 if consumption at or above 40% energy from fat, else 1
13	Coffee	Coffee	Beneficial	1 if consumption at or above the sex-specific median, else 0
14	Green tea	Not available in the dataset	Beneficial	1 if consumption at or above the sex-specific median, else 0

Table S2. Component and scoring of the Healthy Eating Index-2020 (HEI-2020) for ages 2 and over [a] [15]

**Components**	**Maximum points**	**Standard for maximum score**	**Standard for minimum score of zero**
**Adequacy Components**			
Total Fruits [b]	5	≥ 0.8 cup equiv. per 1,000 kcal	No Fruit
Whole Fruits [c]	5	≥ 0.4 cup equiv. per 1,000 kcal	No Whole Fruit
Total Vegetables [d]	5	≥ 1.1 cup equiv. per 1,000 kcal	No Vegetables
Greens and Beans	5	≥ 0.2 cup equiv. per 1,000 kcal	No Dark Green Vegetables or Legumes
Whole Grains	10	≥ 1 .5 oz equiv. per 1,000 kcal	No Whole Grains
Dairy [e]	10	≥ 1.3 cup equiv. per 1,000 kcal	No Dairy
Total Protein Foods [d]	5	≥ 2.5 oz equiv. per 1,000 kcal	No Protein Foods
Seafood and Plant Proteins [f]	5	≥ 0.8 oz equiv. per 1,000 kcal	No Seafood or Plant Proteins
Fatty Acids [g]	10	(PUFAs [h] + MUFAs [i])/SFAs [j] ≥ 2.5	(PUFAs + MUFAs)/SFAs ≤ 1.2
**Moderation Components**			
Refined Grains	10	≤ 1.8 oz equiv. per 1,000 kcal	≥ 4.3 oz equiv. per 1,000 kcal
Sodium	10	≤ 1.1 grams per 1,000 kcal	≥ 2.0 grams per 1,000 kcal
Added Sugars	10	< 6.5% of energy	≥ 26% of energy
Fatty Acids	10	≤ 8% of energy	≥ 16% of energy

a Intakes between the minimum and maximum standards are scored proportionately

b Includes 100% fruit juice

c Includes all forms except juice

d Includes beans, peas, and lentil

e Includes all milk products, such as fluid milk, yogurt, and cheese, and fortified soy beverages

f Includes seafood, nuts, seeds, soy products (other than beverages), and beans, peas, and lentils

g Ratio of poly- and monounsaturated fatty acids (PUFAs and MUFAs) to saturated fatty acids (SFAs)

h PUFAs = polyunsaturated fatty acids

i MUFAS = monounsaturated fatty acids

j SFAs = saturated fatty acids

Table S3. Component and scoring of the Mediterranean Diet Score (MDS) [17]

Component	Scoring
Vegetables	1 if consumption at or above the sex-specific median, else 0
Legumes	1 if consumption at or above the sex-specific median, else 0
Fruits	1 if consumption at or above the sex-specific median, else 0
Cereals	1 if consumption at or above the sex-specific median, else 0
Fish	1 if consumption at or above the sex-specific median, else 0
Meat and poultry	0 if consumption at or above the sex-specific median, else 1
Dairy product	0 if consumption at or above the sex-specific median, else 1
Alcohol consumption	1 to men consuming from 10 g to less than 50 g of ethanol per day and to women consuming from 5 g to 25 g, else 0
Ratio of the sum of monounsaturated and polyunsaturated to saturated fatty acids	1 if consumption at or above the sex-specific median, else 0

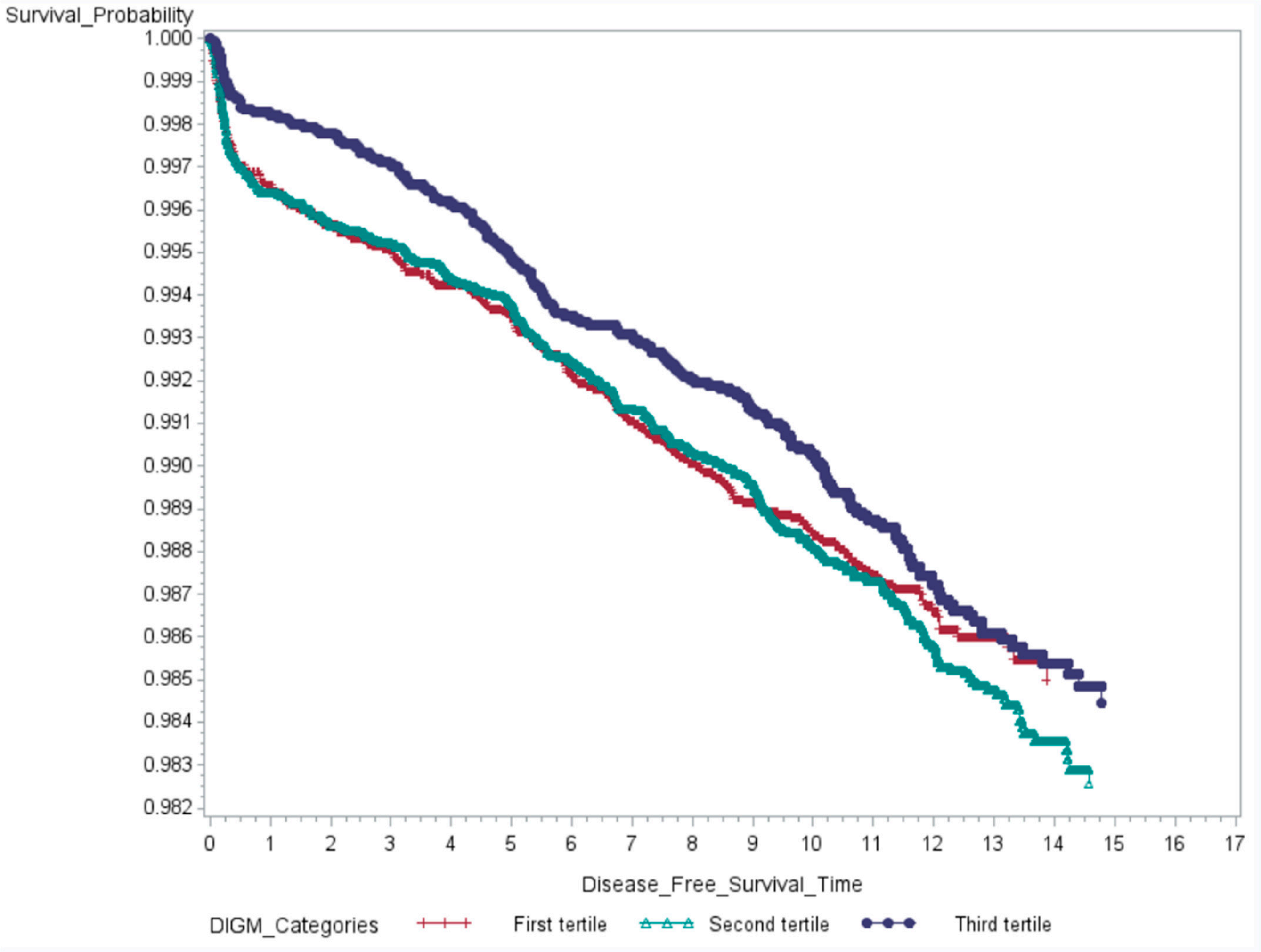

Figure S1. Kaplan-Meier plots of colorectal cancer incidence by DI-GM tertiles in PLCO Study

Table S4. Subgroup analysis of the relationship between dietary indices and colorectal cancer by sex in PLCO

Dietary index	Sex	
	Female (n=27,443)	Male (n=28,242)
Cases (%)	317 (1.2)	418 (1.5)
Person-years	320,204	330,266
**DI-GM** (High versus low quality)[1]		
First 5 years of follow-up	0.73 (0.47, 1.13)	0.88 (0.64, 1.22)
After 5 years of follow-up	1.13 (0.82, 1.57)	0.90 (0.66, 1.23)
**HEI-2020** (High versus low quality)[1]		
First 5 years of follow-up	0.96 (0.67, 1.38)	0.79 (0.57, 1.11)
After 5 years of follow-up	1.16 (0.87, 1.55)	0.85 (0.62, 1.16)
**MDS** (High versus low quality) [1]		
First 5 years of follow-up	0.74 (0.51, 1.06)	0.84 (0.63, 1.11)
After 5 years of follow-up	1.14 (0.85, 1.52)	1.11 (0.84, 1.45)

[1] High quality (above 67th percentile) versus low quality (below 67th percentile), all models are adjusted for age, total energy intake, race, education, marital status, smoking, alcohol use, BMI, regular aspirin or NSAID use, family history of CRC, *P<0.05

Table S5. Subgroup analysis of the relationship between dietary indices and colorectal cancer by tumor location in PLCO

Dietary index	Location of cancer	
	**Distal**	**Proximal**
Cases (%)	310 (0.56)	428 (0.77)
Person-years	647,581	649,271
**DI-GM** (High versus low quality)[1]		
First 5 years of follow-up	0.79 (0.55, 1.12)	0.89 (0.60, 1.31)
After 5 years of follow-up	0.88 (0.57, 1.35)	1.03 (0.79, 1.34)
**HEI-2020** (High versus low quality)[1]		
First 5 years of follow-up	0.90 (0.65, 1.24)	0.86 (0.60, 1.25)
After 5 years of follow-up	0.81 (0.54, 1.21)	1.07 (0.83, 1.37)
**MDS** (High versus low quality) [1]		
First 5 years of follow-up	0.81 (0.60, 1.09)	0.81 (0.57, 1.14)
After 5 years of follow-up	1.26 (0.88 1.81)	1.06 (0.84, 1.34)

[1] High quality (above 67th percentile) versus low quality (below 67th percentile), all models are adjusted for age, total energy intake, sex, race, education, marital status, smoking, alcohol use, BMI, regular aspirin or NSAID use, family history of CRC, *P<0.05

Table S6. Sensitivity analysis of the relationship between dietary indices and colorectal cancer in a sub-sample of PLCO participants with physical activity data (n=40,165)

Dietary index	Multivariable model
Cases (%)	426 (1.1)
Person-years	496,278
**DI-GM** (High versus low quality)[1]	
First 5 years of follow-up	0.78 (0.54, 1.14)
After 5 years of follow-up	1.09 (0.83, 1.44)
**HEI-2020** (High versus low quality)[1]	
First 5 years of follow-up	0.83 (0.59, 1.17)
After 5 years of follow-up	1.07 (0.82, 1.39)
**MDS** (High versus low quality) [1]	
First 5 years of follow-up	0.74 (0.54, 1.02)
After 5 years of follow-up	1.24 (0.97, 1.59)

[1] High quality (above 67th percentile) versus low quality (below 67th percentile), all models are adjusted for age, total energy intake, sex, race, education, marital status, smoking, alcohol use, BMI, regular aspirin or NSAID use, family history of CRC, *P<0.05
